# Choroidal Thickness in Patients with Mild Cognitive Impairment and Alzheimer's Type Dementia

**DOI:** 10.1155/2016/7291257

**Published:** 2016-01-11

**Authors:** Mehmet Bulut, Aylin Yaman, Muhammet Kazim Erol, Fatma Kurtuluş, Devrim Toslak, Berna Doğan, Deniz Turgut Çoban, Ebru Kaya Başar

**Affiliations:** ^1^Antalya Training and Research Hospital, Ophthalmology Department, 07050 Antalya, Turkey; ^2^Antalya Training and Research Hospital, Neurology Department, 07050 Antalya, Turkey; ^3^Department of Animal Science Biometry and Genetics Unit, Faculty of Agriculture, Akdeniz University, 07070 Antalya, Turkey

## Abstract

*Aim*. To asses both choroidal thickness differences among Alzheimer's type dementia (ATD) patients, mild cognitive impairment (MCI) patients, and healthy control (C) subjects and choroidal thickness relationships with cognitive performance.* Methods*. A total of 246 eyes of 123 people (41 ATD, 38 MCI, and 44 healthy C subjects) were included in this study. Complete ophthalmological and neurological examination was performed in all subjects. Choroidal thicknesses (CT) were measured at seven locations: the fovea, 500-1500-3000 *μ*m temporal and 500-1500-3000 *μ*m nasal to the fovea by enhanced depth imaging optical coherence tomography (EDI-OCT). Detailed neurological examination including mini mental state examination (MMSE) test which evaluates the cognitive function was applied to all participants.* Results*. The ages and genders of all participants were similar in all groups. Compared with healthy C subjects, the CT measurements at all regions were significantly thinner both in patients with ATD and in patients with MCI than in healthy C subjects (*p* < 0.05). The MMSE scores were significantly different among ATD patients, MCI patients, and healthy C subjects. They were 19.3 ± 1.8, 24.8 ± 0.9, and 27.6 ± 1.2 in ATD, MCI, and healthy controls, respectively (*p* < 0.001). There were also significant correlation between MMSE score and choroidal thickness at each location (*p* < 0.05).* Conclusions*. CT was reduced in ATD patients and MCI patients. Since vascular structures were affected in ATD patients and MCI patients, they had thin CT. Besides CT was correlated with degree of cognitive impairment. Therefore CT may be a new biomarker in diagnosis and follow-up of MCI and ATD patients.

## 1. Introduction

Alzheimer's type dementia disease (ATD) is the most frequent form of dementia and is characterized by cognitive deficits including progressive memory disturbance, higher cortical functions, executive functions, and other components of cognition [1]. Mild cognitive impairment (MCI) is a recently described syndrome in which patients experience subjective and objective memory deficits or have impairment of other cognitive abilities other than memory. They may yield abnormal scores on memory tests, but their activities of daily living and occupational functions are not affected. MCI represents a clinical condition in which the risk of developing dementia is increased and is accepted as a transitional stage between “healthy” and “dementia” [2]. As human life expectancy continues to increase, the prevalence of ATD and MCI is expected to increase along with the need to treat patients with these diseases [3].

Although ATD is primarily a disease of the brain characterized by cognitive abnormalities, it is also associated with impairments in visual function, including impairments in color perception, depth perception, contrast sensitivity, and visual field [4]. The retina is an extension of the brain that is, like other regions of the brain, derived from the neural tube [5]. Recent reports suggest that degenerative diseases of the retina, including age-related macular degeneration and glaucoma, share common features with ATD. Indeed, ATD, age-related macular degeneration, and glaucoma are all complex, multifactorial forms of degeneration of central nervous system tissue in which age is the primary risk factor and is characterized by protein-aggregate deposition and similar mechanisms for induction of cell injury [6–8].

Clinical studies have indicated that vascular changes play an important role early in ATD pathogenesis, though cerebral amyloid beta (A*β*) plaques and neurodegeneration are the main hallmarks of the disease [9, 10]. Interestingly, both retinal and choroidal vascular A*β* deposits have been observed in animal models of ATD [11]. Although previous studies have reported that choroidal thickness (CT) decreases in ATD patients, none has reported observance of changes in CT in MCI patients [3, 12]. To fill this research gap, this study measured CT in MCI and ATD patients using enhanced depth imaging optical coherence tomography (EDI-OCT) and compared their CT values to those of healthy controls.

## 2. Materials and Methods

The study was approved by the local ethics committee of the site at which it was conducted and performed in accordance with the ethical standards outlined in the Declaration of Helsinki. Informed consent was obtained by all participating subjects prior to their inclusion in the study. 41 Alzheimer's type dementia (ATD) patients, 38 MCI patients, and 44 cognitively healthy age-matched volunteers were enrolled in this study.

The criterion for inclusion for the ATD patients was fulfillment of the National Institute of Neurological and Communicative Disorders and Stroke and the Alzheimer's Disease and Related Disorders Association (NINCDS-ADRDA) criteria. The criteria for inclusion of MCI patients were the absence of other neurological diseases except cognitive impairment or ophthalmologic diseases, the reporting of memory complaints, and an abnormal score on delayed memory recall tests (i.e., two or three missing words), which is a part of mini mental state examination test (MMSE). All the ATD and MCI patients were evaluated by an experienced neurologist. Detailed neurological and mental state examinations and dementia screening tests were completed, supportive laboratory tests were performed, and all the patients were diagnosed clinically as ATD or MCI. Orientation, attention, memory, language, and shape copying are evaluated with MMSE test. Maximum points are 30. For Turkish society the study for validity and reliability was done by Güngen and his associates in 2002 and the cut-off value was determined as 23/24 [13]. This test is considered reliable for identifying the degree of mild dementia. The criterion for inclusion of control subjects was the absence of cognitive impairment and other neurological or ophthalmologic diseases. None of the subjects in the control group had subjective memory complaints. These healthy subjects in the control group also were screened for dementia and underwent a neurological examination by the same neurologist. The exclusion criteria for all groups of patients were having a history or showing evidence of other neurologic or psychiatric disorders, other types of dementia except ATD, retinal disease (i.e., macular degeneration), glaucoma, ocular trauma, ocular surgery, ocular inflammation refractive errors outside −5 to +3 D, diabetes mellitus, systemic arterial hypertension, cardiovascular disease, or other serious chronic systemic diseases, and currently being a smoker.

All subjects underwent optical coherence tomography (OCT) measurement, a complete ophthalmic examination that included assessment of visual acuity and intraocular pressure, slit lamp biomicroscopy, visual field examination, and axial length measurement with optical biometry (Lenstar LS 900, Haag-Streit AG, Köniz, Switzerland).

**Figure 1:**
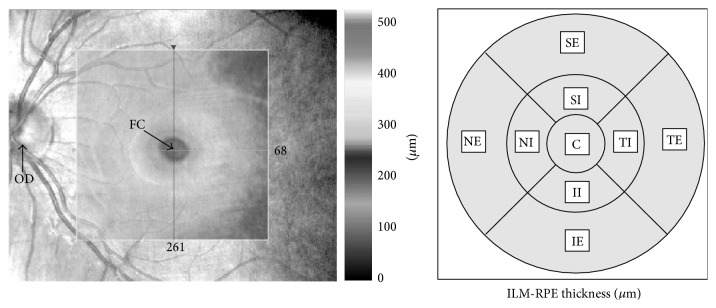
The map was divided into nine ETDRS macular fields. C: central field; SI: superior internal field; TI: temporal internal field; II: inferior internal field; NI: nasal internal field; SE: superior external field; TE: temporal external field; IE: inferior external field; NE: nasal external field.

**Figure 2:**
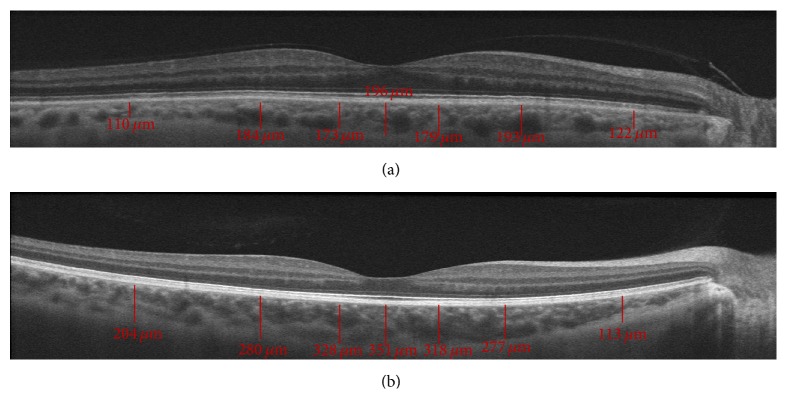
This figure shows that choroidal thickness measurement at each location in both a healthy individual (b) and a patient with ATD (a).

Spectral-domain OCT (SD-OCT) imaging was performed using the Cirrus HD OCT system (Cirrus Carl Zeiss Meditec Inc., Dublin, CA, USA) using the macular cube 512 × 128 protocol, according to which retinal thickness was quantitatively measured in each of the nine regions (Figure 1). EDI-CT was performed by two technicians blind to the patients' diagnoses using the Cirrus HD-OCT Model 5000 (Carl Zeiss Meditec Inc., Dublin, CA, USA). Choroidal thickness imaging was performed by the same independent technician using EDI-OCT following a previously described technique [14]. The participants were asked not to consume caffeine for at least 12 h before examination. Three consecutive measurements of CT at each localization were performed over three days and the average values were calculated. All scans were required to have a signal strength of at least 6/10 to be included in the data analysis. Two technicians blind to the patients' diagnoses measured the perpendicular choroidal thickness from the outer edge of the hyperreflective retinal pigment epithelium to the inner sclera at seven locations: at the fovea; at 500, 1500, and 3000 microns temporal to the fovea; and at 500, 1500, and 3000 microns nasal to the fovea (Figure 2). Three consecutive measurements were taken at each location and the average value was calculated.

The data obtained from the eyes were used for the statistical analysis. One-way analysis of variance analysis (ANOVA) with Bonferroni correction and Spearman correlation analysis were performed using SPSS ver. 20 (SPSS Inc., Chicago, IL, USA). Descriptive statistics were presented in terms of the mean ± standard deviation.* p* value less than 0.05 was accepted as significant.

## 3. Results

**Figure 3:**
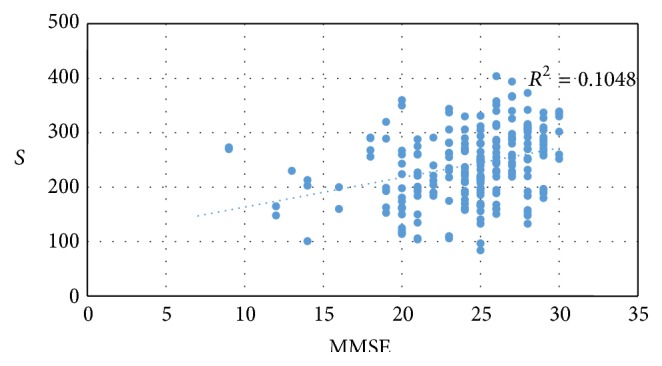
This figure shows that correlation between MMSE scores and subfoveal choroidal thickness.

Table 1 shows the demographic and clinical characteristics of the patients. As can be observed, no significant differences were found among the three groups regarding age, sex, intraocular pressure, and axial length measurements. Tables 2, 3, and 4 and Figure 3 show the results of the data analysis.  Table 2 shows that significant differences were found among the three groups regarding CT at each location, with the ATD and MCI patients found to have the lowest CT values and the control subjects found to have the highest CT value.  Table 3 and Figure 3 show that a significant correlation was also found between MMSE score and CT at each location. Although the macular thickness values of the ATD patients were lower than that of the MCI patients and control subjects at each location, the differences among these values were not found to be statistically significant (Table 4).

## 4. Discussion

Recent research reflects an increased effort to identify a new visual biomarker that can be used to diagnose ATD patients early in the disease process and then to follow the disease process. As MCI patients are considered to have an early form of ATD, it is believed that identification of such a biomarker would assist in their diagnosis and treatment as well. In our attempt to identify such a biomarker, we observed thinning of CT at all localizations in the sample of ATD and MCI patients that we studied. As one of the most vascularized tissues of the body, the choroid functions to provide oxygen and nutrition to the external retinal tissue. Thus, thinning of choroidal tissue in ATD and MCI patients may be related to hypoperfusion and changes due to atrophy, along with the thinning associated with aging [15].

Our findings support previous observation of CT thinning in ATD patients in several recent studies [3, 12]. To our knowledge, the current study was the first in which the thinning of choroidal tissue was observed in MCI patients as well as ATD patients. Accumulation of A*β* and development of neurofibrillary tangles (NFTs) are responsible for the pathogenesis of ATD. Both of these substances first cause neurotoxicity, neuronal and synaptic loss, and vascular angiopathy [4]. In previous studies a relationship between vascular changes and the neurodegenerative process in the clinical risk and progression of ATD was discovered [16, 17]. In accordance with the knowledge that cerebral vascular damage in ATD patients plays a vital role in the disease pathogenesis, it was also observed that cerebral amyloid angiopathy resulted from cerebral arterioles and accumulation of A*β* in the capillaries [18–20]. It has been also observed that accumulation of soluble A*β* causes an increase in vascular resistance of the brain cortex in mice as well as cerebral hypoperfusion and vasoconstriction [21]. It is believed that all these cerebral vascular changes cause oxidative stress and neurotoxicity before clinical dementia starts [18–21].

Both A*β* accumulation and NFTs are found in many parts of the visual system in ATD patients, including the retina [22, 23]. In a mouse model of ATD, A*β* deposits were found in the retina, specifically in the retinal ganglion cells (RGCs), consistent with the pathology observed in the brain [24]. Several researchers have also observed accumulation of A*β* in choroidal vascular tissue in normal aging mice as well as in a mouse model of ATD. Based on their findings, these researchers suggest that like the development of angiopathy in the brain is due to the accumulation of A*β*, it can cause angiopathy in the choroid and, consequently, atrophy [24, 25].

OCT studies seeking to identify a new visual biomarker for early diagnosis and with which to follow the progression of disease in AD and MCI patients have primarily focused on retinal nerve fiber layer (RNFL) and retinal ganglion cell layer (RGCL) thickness. Thinning of RNFL and RGCL thickness is determined in relation to the neurodegenerative period that plays a role in pathogenesis [26–29]. However, few studies have examined the role of CT in this process. To our knowledge, only two previous studies observed lower CT values in AD patients compared with healthy controls [3, 12]. Our study was the first to identify not only a significant decrease in CT values in ATD patients at all locations compared to healthy control subjects, a finding consistent with previous studies, but also a significant decrease in CT values in MCI patients compared with healthy controls.

As MCI has a similar pathogenesis as ATD and is accepted as the early phase of ATD, it was not surprising that we obtained similar findings in the ATD and MCI patients. Based on our findings, we hypothesize that the thinning of choroidal thickness in both ATD and MCI patients is related to the toxicity caused by A*β* accumulation. As previously discussed, A*β* accumulation can cause angiopathy of the cerebral vascular tissue in ATD patients, which leads to causing atrophy. As choroidal tissue is mostly vascular tissue and ATD and MCI have a similar pathogenesis, the accumulation of A*β* may cause angiopathy in a similar way in both AD and MCI patients. This angiopathy will in turn cause atrophy of choroidal tissue, reflected in a reduction in CT values.

In our study, we separately observed a significantly positive correlation between the CT values at all localizations and the MMSE scores, which assess cognitive state. In contrast, Bayhan et al. found no correlation between CT values and MMSE score in their study. Although we observed a decrease in the macular thickness values in AD and MCI patients compared to healthy controls, the differences in the values among these groups were not statistically significant. Previous findings regarding these values are mixed; while some observed thinning in macular thickness in AD patients, others observed no thinning [12, 26, 28].

Our study faced an important limitation that should be considered when evaluating the findings. This limitation was our measurement of CT manually using EDI-OCT, which we believe that it can cause errors in measurement. To help overcome this limitation, we designed the study so that two technicians blind to the patients' diagnoses performed the measurements. We then used the mean values of three different measurements at each localization taken over three days in the data analysis. We believe that the ability to automatically perform CT measurement using new software in the swept source- (SS-) OCT format will greatly decrease measurement error, and hence more fully overcome this limitation, in the future.

In conclusion, we observed that CT values at all locations decrease in both ATD and MCI patients compared to those of healthy controls and identified a positive correlation between MMSE score and CT value. Based on our findings, we propose measurement of CT value using noninvasive, easily performed methods, such as SD-OCT, and use of this value as a new biomarker for early diagnosis of AD and MCI patients, follow-up of progression, and evaluation of the efficiency of new-generation medicines used in their treatment.

## Figures and Tables

**Table 1:** Demographic and clinical characteristics of the study subjects.

	ATD patients (*n* = 82 eyes)	MCI patients (*n* = 76 eyes)	Control subjects (*n* = 88 eyes)	*p* value
Age	73.34 ± 7.0	71.68 ± 7.4	70.65 ± 7.5	0.874
Sex (M/F)	20/21	19/19	21/23	0.752
IOP	14.9 ± 2.7	15.3 ± 2.6	15.2 ± 2.4	0.783
AXL	22.95 ± 1.9	23.02 ± 1.9	23.1 ± 2.1	0.678
MMSE score	19.39 ± 18	24.84 ± 0.9	27.64 ± 1.2	**<0.001**

ATD: Alzheimer's type disease; MCI: mild cognitive impairment; AXL: axial length; IOP: intraocular pressure; and MMSE: mini mental state examination.

**Table 2:** Relationship of choroidal thickness with Alzheimer's type dementia (ATD) and mild cognitive impairment (MCI).

Choroidal thickness (*μ*m)	ATD (*n* = 82)	MCI (*n* = 76)	Controls (*n* = 88)	^*∗*^ *p* value	ATD versus controls		MCI versus controls		ATD versus MCI	
	Mean thickness (SD)	Mean thickness (SD)	Mean thickness (SD)		Mean difference (SE)	^†^ *p* value	Mean difference (SE)	^†^ *p* value	Mean difference (SE)	^†^ *p* value
Subfoveal	215.6 ± 64.5	228.7 ± 52.9	272.7 ± 60.1	**<0.001**	−57.1 (9.4)	**<0.001**	−44.0 (9.6)	**<0.001**	−13.1 (9.9)	0.187
Temporal 0.5 mm	200.4 ± 60.0	211.0 ± 49.9	250.2 ± 55.3	**<0.001**	−49.7 (8.7)	**<0.001**	−39.2 (8.9)	**<0.001**	−10.5 (9.2)	0.253
Nasal 0.5 mm	194.9 ± 63.4	204.7 ± 53.6	244.2 ± 60.5	**<0.001**	−49.3 (9.4)	**<0.001**	−39.5 (9.6)	**<0.001**	−9.7 (9.8)	0.324
Temporal 1.5 mm	192.5 ± 59.3	198.3 ± 47.9	239.0 ± 53.4	**<0.001**	−46.5 (8.5)	**<0.001**	−40.7 (8.6)	**<0.001**	−5.7 (8.9)	0.519
Nasal 1.5 mm	171.3 ± 64.3	182.7 ± 59.7	216.6 ± 68.4	**<0.001**	−45.3 (10.2)	**<0.001**	−33.9 (10.4)	**0.001**	−11.3 (10.7)	0.289
Temporal 3 mm	172.1 ± 50.1	174.4 ± 48.0	210.2 ± 43.9	**<0.001**	−38.1 (7.4)	**<0.001**	−35.8 (7.6)	**<0.001**	−2.2 (7.8)	0.772
Nasal 3 mm	116.5 ± 39.2	120.0 ± 48.3	141.7 ± 52.8	0.002	−21.7 (7.5)	0.004	−25.2 (7.6)	0.001	3.5 (7.9)	0.657

SD: standard deviation, SE: standard error, ^*∗*^ *p* value represents comparison among three groups, and ^†^ *p* value represents the pairwise comparison between two groups.

ANOVA.

Post hoc Bonferroni test.

**Table 3:** Correlation between MMSE score and choroidal thickness at each location.

Choroidal region	*p* value	*r* value
Subfoveal	**<0.001**	0.324
Temporal 0.5 mm	<**0.001**	0.318
Nasal 0.5 mm	<**0.001**	0.298
Temporal 1.5 mm	<**0.001**	0.285
Nasal 1.5 mm	<**0.001**	0.281
Temporal 3 mm	<**0.001**	0.255
Nasal 3 mm	**0.013**	0.164

Results obtained by Spearman correlation test.

**Table 4:** Comparison of macular thickness among the study groups.

Macular thickness (*μ*m)	ATD (*n* = 82)	MCI (*n* = 76)	Controls (*n* = 88)	^*∗*^ *p* value
	Mean thickness (SD)	Mean thickness (SD)	Mean thickness (SD)	
Average	271.3 ± 15.6	275.8 ± 13.6	276.5 ± 14.0	0.070
C	248.2 ± 26.7	252.5 ± 29.5	254.6 ± 27.1	0.337
SE	274.0 ± 15.2	274.0 ± 20.4	275.6 ± 15.9	0.802
NE	289.6 ± 15.0	291.9 ± 19.6	293.7 ± 15.6	0.320
IE	262.1 ± 19.6	264.3 ± 18.7	265.4 ± 15.0	0.481
TE	257.5 ± 17.8	258.9 ± 17.1	261.0 ± 15.4	0.450
SI	310.1 ± 38.3	318.3 ± 22.0	319.2 ± 19.3	0.081
NI	315.8 ± 17.8	320.2 ± 21.9	321.3 ± 18.7	0.178
II	312.6 ± 20.3	315.4 ± 21.2	317.4 ± 19.2	0.324
TI	303.6 ± 19.0	308.4 ± 21.3	309.3 ± 19.1	0.159

Results obtained by one-way ANOVA test. ATD: Alzheimer's type disease; MCI: mild cognitive impairment; SD: standard deviation, C: central field; SI: superior internal field; TI: temporal internal field; II: inferior internal field; NI: nasal internal field; SE: superior external field; TE: temporal external field; IE: inferior external field; and NE: nasal external field.

^*∗*^ *p* value represents comparison among the three groups.
